# Activity of temocillin against third-generation cephalosporin-resistant *Escherichia coli* and *Klebsiella pneumoniae* bloodstream isolates from a clinical trial

**DOI:** 10.1093/jacamr/dlab192

**Published:** 2021-12-28

**Authors:** Adam G Stewart, Andrew Henderson, Michelle J Bauer, David L Paterson, Patrick N A Harris

**Affiliations:** 1 Centre for Clinical Research, Faculty of Medicine, The University of Queensland, Royal Brisbane and Women’s Hospital Campus, Brisbane, Australia; 2 Department of Infectious Diseases, Royal Brisbane and Women’s Hospital, Brisbane, Australia; 3 Central Microbiology, Pathology Queensland, Royal Brisbane and Women’s Hospital, Brisbane, Australia; 4 Infection Management Services, Princess Alexandra Hospital, Brisbane, QLD, Australia

## Abstract

**Background:**

Extended spectrum β-lactamase (ESBL) and AmpC-producing Gram-negative bacilli contribute significantly to the antimicrobial resistance (AMR) burden worldwide. Temocillin is an intravenous semisynthetic antibiotic that is stable to hydrolysis by ESBLs and AmpC. Temocillin may be a treatment option for serious infections due to these organisms.

**Methods:**

Third-generation cephalosporin-resistant *Escherichia coli* and *Klebsiella pneumoniae* isolates from the MERINO trial were collected. The majority originated from the urinary tract. Isolates had previously undergone whole genome sequencing (WGS) to identify antimicrobial resistance genes. Temocillin minimum inhibitory concentration (MIC) values were determined by broth microdilution (BMD) with a concentration range of 2 to 128 mg/L. A recent EUCAST guideline has recommended clinical breakpoints for urinary *E. coli*, *Klebsiella* spp. (except *K. aerogenes*) and *Proteus mirabilis* (resistant >16 mg/L).

**Results:**

317 index bloodstream isolates (275 *E. coli* and 42 *K. pneumoniae*) were used. The frequency of β-lactamases among isolates was: CTX-M-15 (56%), OXA-1 (31%), CTX-M-27 (14%), CTX-M-14 (12%) and CMY-2 (8%). Overall, 95% of isolates were susceptible, increased exposure according to EUCAST clinical breakpoints v11.0. Summary MIC values were obtained: MIC_50_ was 8 mg/L and MIC_90_ was16 mg/L (range ≤2 to ≥128 mg/L) and did not differ markedly between species. Higher MIC values were seen among isolates that produced more than one β-lactamase but this did not appear to be specific to a single β-lactamase.

**Conclusions:**

Temocillin demonstrated favourable *in vitro* activity against ceftriaxone-resistant Enterobacterales bloodstream isolates and may be a suitable agent to be trialled for treatment of serious infections due to these organisms.

## Introduction

β-Lactamase enzymes produced by Gram-negative bacilli (GNB) are a common mechanism of antimicrobial resistance (AMR).^1^ Extended-spectrum β-lactamases (ESBLs) and AmpC β-lactamases are capable of hydrolysing commonly used broad-spectrum antibiotics such as ceftriaxone, cefotaxime and ceftazidime.^1^ Carbapenems are considered the first-line treatment option for severe infections due to ESBL- and AmpC-producing Enterobacterales. In no observational study has the outcome of serious infections for ESBL or AmpC producers treated with carbapenems been significantly surpassed by any other agents.^2–4^ Moreover, a landmark clinical trial comparing piperacillin/tazobactam with meropenem in the treatment of bloodstream infection due to ESBL-producing *Escherichia coli* and *Klebsiella pneumoniae* showed that piperacillin/tazobactam was not non-inferior to meropenem with respect to 30 day all-cause mortality.^5^ Widespread use of carbapenem antibiotics may lead to a rising incidence of carbapenem-resistant GNB.^6^ Availability of a carbapenem-sparing antimicrobial for the treatment of ESBL and AmpC producers would be of considerable value in halting the emergence of carbapenem resistance.

Temocillin is a semisynthetic 6-α-methoxylpenicillin antibiotic, derived from ticarcillin, developed in the 1980s and available for parenteral administration.^7^ It demonstrates stability against β-lactamases including extended-spectrum SHV/TEM variants and CTX-M.^8^ This property has renewed the interest in this antimicrobial as a potential carbapenem-sparing therapy. Temocillin is currently registered for use in several European countries but is yet to be introduced in Australia or the United States. Recent changes to temocillin clinical breakpoints have been proposed by EUCAST.^9^ Here we assess the *in vitro* activity of temocillin against third-generation cephalosporin-resistant *E. coli* and *K. pneumoniae* bloodstream isolates obtained from a clinical trial.

## Materials and methods

### Bacterial isolates

The MERINO trial recruited patients with bloodstream infection due to third-generation cephalosporin non-susceptible *E. coli* and *K. pneumoniae* in nine countries from February 2014 to July 2017.^5^ All index blood culture isolates from patients meeting the inclusion criteria who consented to participate in the study were stored at the recruiting site laboratory at −80°C and later shipped to the coordinating laboratory in Queensland, Australia (University of Queensland Centre for Clinical Research). Stored isolates had previously undergone whole genome sequencing (WGS) to detect antimicrobial resistance genes.^5^^,^^10^ Each isolate was subjected to broth microdilution (BMD) testing for temocillin minimum inhibitory concentration (MIC) determination.

### BMD preparation

BMD was performed according to ISO 20776-1 for non-fastidious organisms in unsupplemented Mueller-Hinton broth.^11^ Custom-made Sensititre plates sourced from Thermo Fisher Scientific were used and incorporated temocillin with a concentration range from 2 to 128 mg/L. This range was chosen to include the EUCAST breakpoint for *E. coli* and *Klebsiella* spp. (except *K. aerogenes*) of urinary origin; resistant >16 mg/L. The wild-type population for these species have been placed in the intermediate group (1–16 mg/L) with 16 mg/L representing the epidemiological cut-off values (ECOFFs) for most target species.^9^ This implies that EUCAST do not specify a ‘Susceptible’ category and emphasizes the use of high dose temocillin (2 g q8h) for urinary tract isolates with MIC <16 mg/L. Prepared antibiotic was dispensed into custom made Sensititre labelled 96-well plates (ThermoScientific, 262162) which were inoculated. *Escherichia coli* ATCC 25922, target MIC 16 mg/L was used to check the performance of each batch of trays. Test and reference isolates were stored in brain heart infusion (BHI) broth (BD, Bacto 237500) containing 30% glycerol (Chem-Supply, GA010) at −80°C. Each well containing antibiotic was inoculated with 50 μL of broth (5 × 10^5^ cfu/mL). Purity and colony count checks were also performed.

## Results

**Figure 1.**
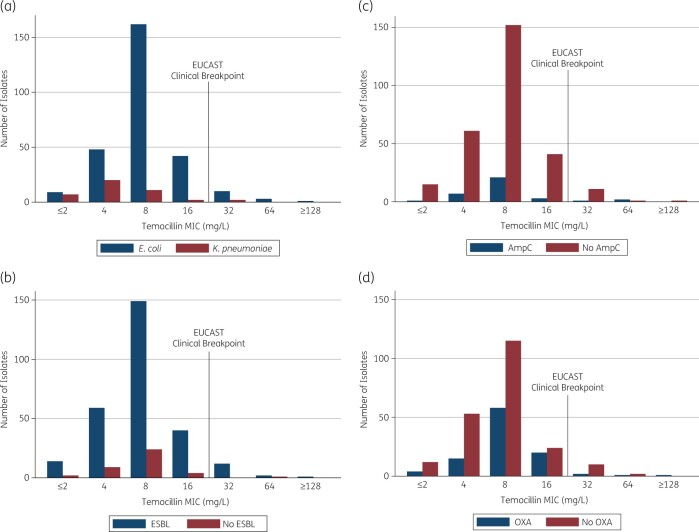
Temocillin MIC values determined by broth microdilution (BMD) of 317 *E. coli* and *K. pneumoniae* bloodstream isolates by (a) species, (b) ESBL, (c) AmpC β-lactamase, and (d) narrow-spectrum OXA β-lactamase.

A total of 317 bloodstream isolates (275 *E. coli* and 42 *K. pneumoniae*) from the MERINO trial were collected and had their temocillin MIC determined by BMD. The most frequent β-lactamase types observed among isolates included CTX-M-15 (178/317, 56%), OXA-1 (98/317, 31%), CTX-M-27 (43/317, 14%), CTX-M-14 (37/317, 12%) and CMY-2 (24/317, 8%) (Tables 1 and 2). Overall, 95% of isolates had MIC values ≤16 mg/L, thus falling into the susceptible, increased exposure ranges according to EUCAST. Summary MIC values were obtained: MIC_50_ was 8 mg/L and MIC_90_ was 16 mg/L (range ≤2 to ≥128 mg/L). *K. pneumoniae* isolates appeared to have lower MIC values when compared with *E. coli* (MIC_50_ values 4 and 8 mg/L, respectively). Interestingly, similar MIC values were seen among isolates producing ESBLs and AmpC β-lactamases (Figure 1). Producing a combination of β-lactamases, whether ESBL, AmpC or narrow-spectrum OXA, conferred higher temocillin MIC values. In particular, *E. coli* isolates that co-produced CTX-M type and CMY β-lactamases had high temocillin MIC values (Table 1). The MIC values for all the plates testing ATCC strains fell within acceptable ranges. Purity and colony count checks demonstrated pure growth and colony counts ranging from 1–9 colonies.

**Table 1.** Temocillin MIC frequency distribution for 275 *E. coli* bloodstream isolates according to β-lactamase type

Organism characteristics	Number of isolates with MIC (mg/L):								Susceptible, increased exposure (%) by EUCAST clinical breakpoint
	≤2	4	8	16	32	64	≥128	Total no.	
All organisms	9	48	162	42	10	3	1	275	95
No ESBL, AmpC or narrow-spectrum OXA		3	6	1				10	100
ESBL only (*n = *149)									
CTX-M-3			2					2	100
CTX-M-14	4	8	15	4	1			32	97
CTX-M-15	1	8	36	13	4			62	94
CTX-M-24			1					1	100
CTX-M-27	2	13	25	1	2			43	95
CTX-M-55		2	5	2				9	100
CTX-M-134					1			1	0
CTX-M-174			1					1	100
Narrow-spectrum OXA only (*n = *1)									
OXA-1			1					1	100
AmpC only (*n = *27)									
CMY-2		4	14	2		1		21	95
CMY-42		1	1					2	100
CMY-146			1					1	100
DHA-1	1		1	1				3	100
ESBL+narrow-spectrum OXA (*n = *83)									
CTX-M-15 + OXA-1	1	8	48	18	1	1	1	78	96
CTX-M-15 + OXA-10			2					2	100
CTX-M-14 + OXA-1		1						1	100
CTX-M-3 + OXA-1			1					1	100
SHV-12 + OXA-9		1						1	100
ESBL + AmpC (*n = *4)									
CTX-M-55 + CMY-2			1			1		2	50
CTX-M-15 + CMY-138			1		1			2	50
ESBL + AmpC + narrow-spectrum OXA (*n = *2)									
CTX-M-15 + CMY-2 + OXA-1			1					1	100
CTX-M-15 + CMY-138 + OXA-1			1					1	100

**Table 2.** Temocillin MIC frequency distribution for 42 *K. pneumoniae* bloodstream isolates according to β-lactamase type

Organism characteristics	Number of isolates with MIC (mg/L):								Susceptible, increased exposure (%) EUCAST clinical breakpoint
	≤2	4	8	16	32	64	≥128	Total no.	
All organisms	7	20	11	2	2	0	0	42	95
No ESBL, AmpC or narrow-spectrum OXA ESBL only (*n* = 23)	1							1	100
CTX-M-3		1	1					2	100
CTX-M-14	1		2					3	100
CTX-M-15	2	12	3		1			18	94
AmpC only (*n = *1)									
DHA-1		1						1	100
ESBL + narrow-spectrum OXA (*n = *15)									
CTX-M-15 + OXA-1	3	5	4	2	1			15	93
ESBL + AmpC (*n = *2)									
CTX-M-14 + DHA-1		1						1	100
SHV-106 + DHA-1			1					1	100

## Discussion

Overall, temocillin demonstrated favourable *in vitro* potency against ESBL-, AmpC- and narrow-spectrum OXA-producing *E. coli* and *K. pneumoniae*. Of note, higher temocillin MICs were observed among isolates harbouring multiple β-lactamases (e.g. ESBL and AmpC). The presence of a specific β-lactamase did not appear to directly influence the MIC. For example, the addition of narrow-spectrum OXA to CTX-M-type β-lactamase did not appear to affect the MIC. Similarly, those isolates harbouring only AmpC β-lactamases did not have higher MIC values when compared with those with only CTX-M-type β-lactamases. The EUCAST breakpoint tables v11.0 (January 2021) have incorporated novel species-specific [*E. coli*, *Klebsiella* spp. (excluding *K. aerogenes*), and *P. mirabilis*] MIC breakpoints for temocillin: S ≤ 0.001, R > 16 mg/L. EUCAST have stated that having an ‘R’ breakpoint below 16 mg/L would split the wild-type of the relevant species that are believed to be good targets for temocillin. Temocillin dosing of 2 g q8 h for urinary isolates with an MIC of ≤16 mg/L is recommended.^12^ 95% of all isolates tested in our study had MICs below the EUCAST clinical breakpoint for resistance. Previously, the British Society of Antimicrobial Chemotherapy (BSAC) guidelines defined temocillin MIC breakpoints for Enterobacteriaceae as susceptible if ≤8 mg/L in systemic infections and ≤32 mg/L in urinary tract infections.^13^ Under those guidelines, 81% and 99% of our tested isolates would have been regarded as susceptible, respectively.

*In vitro* activity of temocillin against MDR GNB has been examined in different settings worldwide. Among 177 ESBL-producing Enterobacterales from Poland, 62% and 94% were susceptible according to BSAC systemic (≤8 mg/L) and urinary tract infection (UTI) (≤32 mg/L) breakpoints, respectively;^14^ MIC_50_ and MIC_90_ values were 8 mg/L and 32 mg/L, respectively. Another study examined 118 ESBL producers from Hong Kong and demonstrated susceptibility rates of 84% and 100%, respectively.^15^ Another study from England tested temocillin against 846 ESBL- and AmpC-producing Enterobacterales and showed susceptibility rates (systemic and UTI) of 63% and 99% for ESBL producers, and 67% and 99% for AmpC producers.^16^ A Norwegian study looked at 105 ESBL-producing *E. coli* and showed rates of 71% and 100%.^17^ Interestingly, one study demonstrated reduced susceptibility to temocillin among AmpC producers when compared with ESBL-producing Enterobacterales.^18^ That same study demonstrated a correlation between the rate of temocillin resistance and the number of β-lactams without *in vitro* activity. The mechanisms of resistance to temocillin in Enterobacterales are largely unknown.^18^ Molecular studies on temocillin resistance mechanisms in *Pseudomonas aeruginosa* identified the role of the MexAB-OprM efflux system, as well as OpdK or OpdF anion-specific porin expression.^19^

Overall, favourable susceptibility results have been seen for UTI, with more variable results for systemic infection. This may reflect the decision by EUCAST to apply current species-related breakpoints to isolates from patients with urinary tract infection only.

Favourable activity of temocillin against an ESBL CTX-M-15 *E. coli* in a murine UTI model has been shown.^20^ Temocillin given at standard doses worked well against *E. coli* strains with an MIC of 16 mg/L or less. There remains limited data on the clinical effectiveness of temocillin in the treatment of infections due to MDR GNB. A study examining 92 infection episodes due to ESBL and AmpC producers demonstrated clinical cure and microbiological cure rates of 86% and 84%, respectively, with use of temocillin.^21^ Similar results were observed in a small case series.^22^ Temocillin may also have a more favourable effect on the gut microbiome. Compared with ceftriaxone, temocillin did not promote expansion of ESBL-producing *E. coli* in faeces of colonized mice.^23^

Key data on the use of temocillin remain unknown. Further *in vitro* and animal model work needs to be performed to better understand the pharmacokinetic and pharmacodynamic properties of the drug. Indeed, clinical data including outcomes with temocillin use will also better inform appropriate clinical breakpoints. Currently there are a few small registered clinical studies either in the recruitment or completion phases comparing temocillin with a carbapenem for treatment of infection due to ESBL- and/or AmpC-producing organisms, including the TEMO-CARB (NCT03543436), TEMO-BLSE (NCT04671290) and TEMO-ESBL (NCT02681263) trials.

### Conclusions

Temocillin demonstrated favourable *in vitro* activity against ESBL- and AmpC-producing Enterobacterales bloodstream isolates obtained from a clinical trial. Summary MIC values were obtained and indicated an MIC_50_ of 8 mg/L and an MIC_90_ of 16 mg/L (range ≤2 to ≥128 mg/L). Temocillin may be an appropriate carbapenem-sparing antibiotic, however, the results of well-designed clinical trials are awaited to determine its utility in clinical practice.
